# 876. Real-world impact of a novel host response test for distinguishing between bacterial and viral infections in pediatric patients at ten Urgent Care Centers

**DOI:** 10.1093/ofid/ofad500.921

**Published:** 2023-11-27

**Authors:** Shirley Shapiro Ben David, Daniella Rahamim-Cohen, Boaz kalmovich

**Affiliations:** Maccabi Healthcare Services, Tel Aviv, Tel Aviv, Israel; Maccabi Healthcare Services, Tel Aviv, Tel Aviv, Israel; Maccabi Healthcare Services, Tel Aviv, Tel Aviv, Israel

## Abstract

**Background:**

Antibiotic misuse is a major driver of antimicrobial resistance and is prevalent in the urgent care setting. A leading cause of misuse is uncertainty regarding infection etiology. MeMed BV® (MMBV) is an FDA-cleared test designed to aid physicians in differentiating between bacterial and viral infections based on computational integration of the blood levels of three host proteins into a score. Here we examined the real-world impact of integrating MMBV in the urgent care workflow on clinical decision-making.

**Methods:**

A retrospective cohort study. MMBV test was ordered as part of the routine care for clinically appropriate pediatric patients at ten Maccabi Healthcare Services outpatient urgent care centers in Israel. MMBV results were interpreted based on manufacturer’s instructions as viral or bacterial infection or equivocal.

As part of MMBV evaluation, physicians who ordered the test were immediately and automatically asked to complete a short questionnaire reporting their initial etiological diagnosis and likelihood to prescribe antibiotics. Upon discharge, physicians completed a second questionnaire to report if MMBV impacted their decision-making process.

The physician’s intent, MMBV results and final antibiotic prescription practice were compared to evaluate the impact of MMBV on antibiotic misuse.

**Results:**

Between 04-12/2022, 2,265 patients were enrolled and 1,759 had fully filled out questionnaires (Figure 1, Table 1). In 1,566 cases, a bacterial or viral MMBV result was received.

Among the 449 cases for whom physicians initially intended to prescribe antibiotics, 320 (71.7%) had viral MMBV results and in 198 cases (61.9%) the physician did not prescribe antibiotics. Conversely, among the 805 cases for whom physicians did not initially intend to prescribe antibiotics, 153 (19%) had bacterial MMBV results and the physician prescribed antibiotics in 93 (60.8%) cases.

Physicians reported uncertainty with regard to antibiotic prescription in 312 (19.9%) cases and acted in accordance with MMBV results in 236 (75.6%). Overall, physicians reported that MMBV aided in their patient management for 82.4% of cases.

**Figure:**
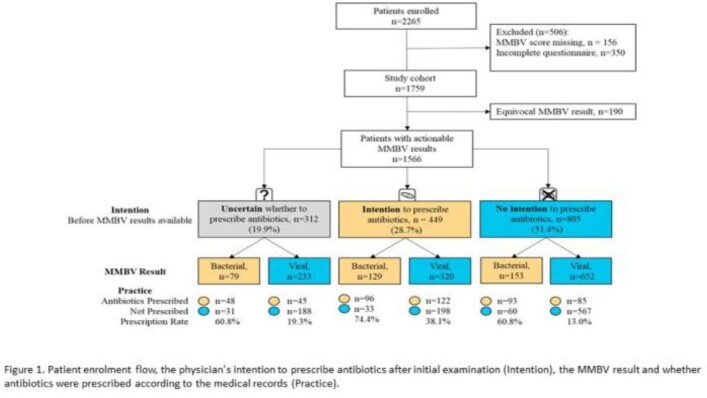

**Figure:**
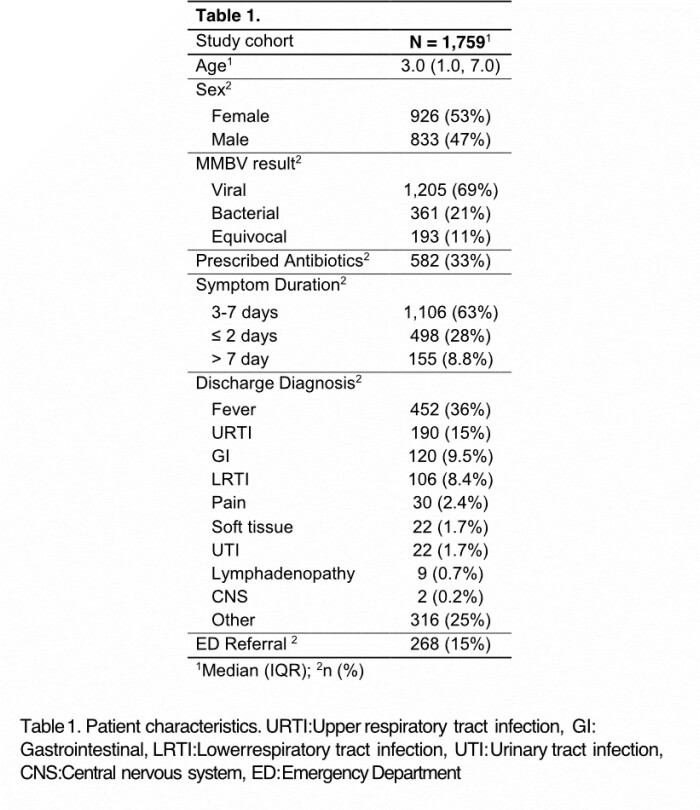

**Conclusion:**

Physicians incorporate MMBV into their real-world decision-making in the urgent care center setting and report that it aids in patient management.

**Disclosures:**

**All Authors**: No reported disclosures

